# Isoform 165 of vascular endothelial growth factor in collagen matrix improves ovine cryopreserved ovarian tissue revascularisation after xenotransplantation in mice

**DOI:** 10.1186/s12958-015-0015-2

**Published:** 2015-03-07

**Authors:** Laurie Henry, Soraya Labied, Maïté Fransolet, Nathalie Kirschvink, Silvia Blacher, Agnès Noel, Jean-Michel Foidart, Michelle Nisolle, Carine Munaut

**Affiliations:** Laboratory of Tumor and Development Biology, Groupe Interdisciplinaire de Génoprotéomique Appliquée (GIGA-R), University of Liège (B23) Sart-Tilman, B-4000 Liège, Belgium; Department of Gynecology, University of Liège, Boulevard du XIIème de Ligne, B-4000 Liège, Belgium; Veterinary Integrated Research Unit, University of Namur, Rue de Bruxelles 61, B-5000 Namur, Belgium

**Keywords:** Collagen matrix, VEGF165, Fertility preservation, Xenotransplantation and angiogenesis

## Abstract

**Background:**

Aggressive anti-cancer treatments can result in ovarian failure. Ovarian cryopreservation has been developed to preserve the fertility of young women, but early graft revascularisation still requires improvement.

**Methods:**

Frozen/thawed sheep ovarian cortical biopsies were embedded in collagen matrix with or without isoform 165 of vascular endothelial growth factor (VEGF165) and transplanted into ovaries of immunodeficient mice*.* Ovaries were chosen as transplantation sites to more closely resemble clinical conditions in which orthotopic transplantation has previously allowed several spontaneous pregnancies.

**Results:**

We found that VEGF165 significantly increased the number of Dextran-FITC positive functional vessels 3 days after grafting. Dextran- fluorescein isothiocyanate (FITC) positive vessels were detectable in 53% and 29% of the mice in the VEGF-treated and control groups, respectively. Among these positive fragments, 50% in the treated group displayed mature smooth-muscle-actin-alpha (alpha-SMA) positive functional vessels compared with 0% in the control group. CD31 positive murine blood vessels were observed in 40% of the VEGF165 transplants compared with 21% of the controls. After 3 weeks, the density of murine vessels was significantly higher in the VEGF165 group.

**Conclusion:**

The encapsulation of ovarian tissue in collagen matrix in the presence of VEGF165 before grafting has a positive effect on functional blood vessel recruitment. It can be considered as a useful technique to be improved and further developed before human clinical applications in female cancer patients in the context of fertility preservation.

## Background

Cancer survival in girls and young women has significantly improved; however, radiotherapy and chemotherapy may alter ovarian function by reducing the primordial follicle reserve. As a result, patient fertility may be impaired and premature ovarian failure (POF) may occur [1-3]. Therefore, techniques for female fertility preservation have been developed, including oocyte, fertilised embryo, and ovarian tissue preservation [2,4]. To date, ovarian tissue preservation remains the optimal procedure for prepubertal girls who, after cancer remission, may be auto-transplanted when the desire for pregnancy arises.

Adult ovarian tissue cryopreservation and transplantation techniques have been successfully performed and have resulted in the birth of 30 children [5,6]. However, this procedure still needs to be optimised because significant follicular loss has been observed during cryopreservation and subsequent grafting [7,8]. Grafting whole ovaries cryopreserved with vascular anastomosis may prevent follicle loss through ischemia. However, this method suffers from a number of drawbacks including a difficult cryopreservation process, a laborious surgical technique, which does not always prevent ischemic damage, and the possibility of reintroducing malignant cells [9,10]. The most important challenges in the transplantation of cryopreserved ovarian fragments are tissue ischemia and reperfusion injury [11,12]. Transient hypoxia during the first 5 days after ovarian tissue transplantation is followed by gradual oxygenation recovery, which is associated with restored angiogenesis and reperfusion over the next 5 days [13,14].

A reduction in post-transplantation hypoxia and subsequent follicular loss could significantly improve ovarian graft efficiency. Therefore, angiogenic factors have been tested in cryopreservation and xenotransplantation models. Sphingosine-1-phosphate (S1P) is a sphingolipid metabolite that decreases apoptosis and stimulates angiogenesis and endothelial cell migration. S1P promotes neoangiogenesis in ovarian transplants and reduces ischemic reperfusion injury during the days following transplantation in a xenograft mouse model [15]. In addition, the use of erythropoietin (EPO) was found to improve early follicle morphology and stromal cell density in canine ovarian transplants after 8 weeks and to enhance blood vessel density 16 weeks after xenografting in severe combined immunodeficient (SCID) mice [16].

The use of growth factors to improve ovarian tissue survival after grafting has progressed beyond the preclinical stage. Callejo et al. reported the first human live birth after impregnation with the frozen/thawed ovarian cortex of the patient and platelet-rich plasma (PRP) [17]. Therefore, it is important to precisely delineate the cytokines that may convey a significant clinical advantage to ovarian grafting efficiency.

Vascular endothelial growth factor (VEGF), specifically VEGF-A, is a potent angiogenic factor that stimulates vascular endothelial cell survival, proliferation, migration, and differentiation. Moreover, some data suggest that VEGF-A appears to be involved in folliculogenesis with the presence of VEGF-A receptors in granulosa cells [18]. VEGF is a disulfide-bonded dimeric glycoprotein that is encoded by a gene that contains 8 exons. VEGF acts mainly through binding to VEGF receptor-2 (VEGF-R2) but also binds to VEGF receptor-1 (VEGF-R1). In addition, alternative splicing creates numerous isoforms, mainly VEGF_121_, VEGF_165_, and VEGF_189_. VEGF_165_ (containing exons 1–5, 7, and 8) is secreted as covalently linked homodimers of 45 kDa with a basic character and moderate affinity for heparin. VEGF_165_ can be found in many tissue types in a soluble form or anchored to the extracellular matrix, and a minimal amount of matrix-bound VEGF_165_ can support endothelial cell growth [19-22]. We previously demonstrated the beneficial effect of VEGF_111_, a highly soluble and diffusible VEGF isoform, in an original model of ewe ovarian tissue xenografts in SCID mice. VEGF_111_ lacks exons 5–7 and is resistant to proteolysis [20]. The use of this isoform in a collagen matrix, which encapsulates the ovarian cortex at the time of transplantation, was found to improve angiogenesis and decrease hypoxia, thereby enhancing the preservation of primary follicles [23].

For this study, sheep ovarian cortex was used due to ethical barriers and the limited availability of human ovarian tissue. Sheep ovary is an adequate candidate for research due to its ovarian architectures (which are similar to those of human ovarian tissue), limited number of developed follicles and single ovulation with primordial follicles distributed superficially in the cortex. Its size is 80% of that of the human premenopausal ovary, and it has a comparable collagen-dense cortical layer containing the pool of primordial follicles [24]. The sheep ovary has been used in many studies [7,25-28].

In this study, using the same xenograft model, we tested whether VEGF_165_, which, unlike isoform 111, is anchored to the extracellular matrix, could improve ovarian graft survival by extending the exposure of the ovarian fragment to this cytokine through sustained release from collagen.

## Methods

### Ovarian tissue sampling

The ethics committee of the University of Liège approved the present study (reference 1156, February 2011). Study was initiated in March 2011 and terminated in October 2013.

Five ewes (10 ovaries) were obtained from the Ovine Research Centre (University of Namur, Belgium). Veterinary euthanised ewes were intravenously injected with T61 1 ml/Kg (MSD, Belgium), a laparotomy was performed, and ovaries were sampled immediately. The ovaries were placed in Leibovitz L-15 medium (Lonza, Basel, Switzerland) supplemented with 10% normal sheep serum (Hormonology Laboratory, Belgium) and kept at 4°C during transport (approximately 1 hour (h)) and during the preparation (approximately 1 h) of cortical fragments.

### Cryopreservation and thawing processes

As previously described [29], ovarian cortical fragments were prepared by cutting the cortex into pieces (2.5 × 2.5 × 1 mm) after the medullar tissue was resected. These ovarian strips were equilibrated in cryopreservative medium that contained Leibovitz L-15 medium, which was supplemented with dimethyl sulfoxide 1.5 M (DMSO) (Sigma), 10% normal sheep serum, and 0.1 M sucrose for 30 min at 4°C. They were transferred into cryovial tubes (Simport Scientific, Canada) containing the same medium. The tubes were cooled in a programmable freezer (CL-8800i System, Cryologic Pty., Ltd., Australia) and stored in liquid nitrogen [25]. Ovarian samples were stored in cryotubes for several weeks to several months until transplantation into mice (minimum 2 weeks, maximum 6 months). The cryovial tubes were kept at room temperature (RT) for 2 min to thaw and then immersed in a 37°C water bath until the frozen media melted, approximately 2 to 3 min. After thawing, the ovarian strips were given three 5 min washes at 37°C in Leibovitz medium to remove the cryoprotective agents.

### Ovarian transplant encapsulation and SCID mouse transplantation

**Figure 1:**
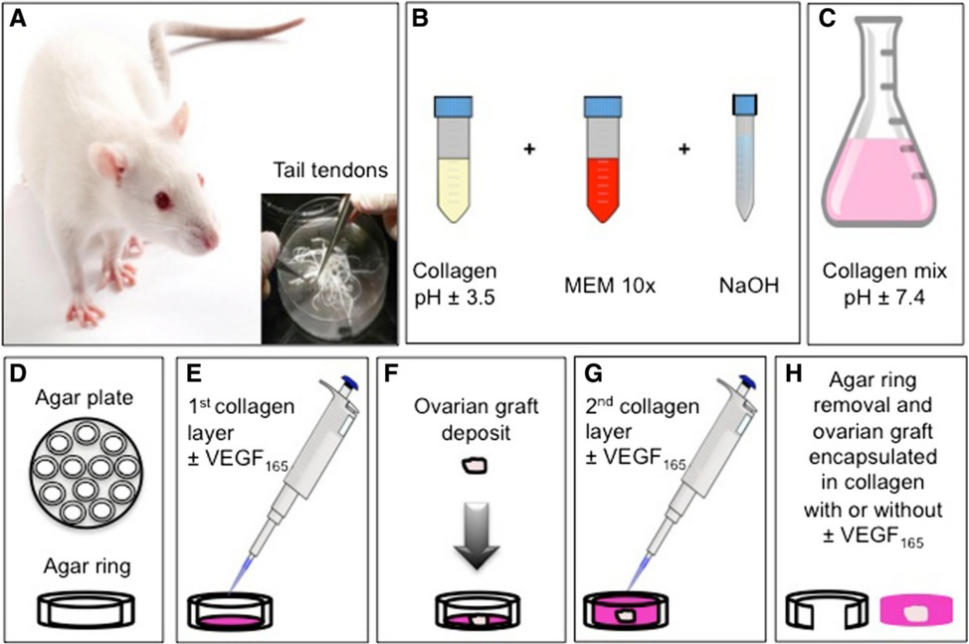
**Encapsulation of ovarian graft in collagen matrix with and without VEGF**_**165**_ **. A**. Type I collagen was extracted from rat tail tendons. **B**. Preparation of collagen matrix. **C**. Adjusting collagen mix to pH 7.4. **D**. Illustration of agarose rings. **E**. Filling of agarose ring with a first layer of the collagen matrix with or without recombinant murine VEGF_165._ **F**. Frozen/thawed ovarian sheep explant deposit. **G**. Second layer of collagen matrix with or without recombinant murine VEGF_165._ **H**. Agar ring removal before transplantation.

Ewe ovarian cortical strips were encapsulated in a three-dimensional type I collagen (Col I) matrix with or without VEGF_165_ as previously described [23] and illustrated in Figure 1. Collagen matrix was prepared by mixing 9 volumes of Col I (2.4 mg/ml) extracted from rat tail tendons [30], 1 volume of 10X minimal essential medium (Life Technologies, Belgium) and approximately 0.1 volume of 1 M NaOH to adjust the pH to 7.4 (Figures 1A-B). Agarose rings (1.5% Agarose VII solution, Sigma, Belgium) were filled with the first layer of collagen (60 μL) matrix with or without recombinant murine VEGF_165_ (50 nM, Peprotech, Rocky Hill NJ, USA, Cat N° 450–32). After polymerisation, frozen/thawed ovarian sheep explant (2.5 × 2.5 × 1 mm) was placed and overlaid by a second layer of Col I (60 μL). The agar ring was removed before transplantation in mice (Figures 1C-G).

Eight-week-old female SCID mice (n = 66, Charles River, Burlington MA, USA) were selected for ovarian xenotransplantation because the SCID affected both B and T lymphocytes (Table 1). After shaving and anaesthesia by intraperitoneal injection of ketamine (100 mg/kg) and xylazine (10 mg/kg), a dorsal incision was performed and a single ovarian cortex, which was encapsulated in collagen gel, was stitched into the right mouse ovary using 7–0 Prolene sutures. To detect functional vessels and possible anastomoses of sheep and mouse vessels in the grafted ovary transplant, 200 μl of dextran-fluorescein isothiocyanate (FITC, 2.5 mg/ml in PBS) (Sigma-Aldrich, Saint Louis, MO, USA) was intravenously injected 3 min before sacrifice. Functional vessels were detected on transplanted sections with anti-fluorescein Ab (Converter-POD, Roche, Penzberg, Germany). The animals were euthanized 3 days or 3 weeks after grafting. Ovarian cortical tissue specimens were recovered and fixed in 4% formaldehyde.

**Table 1:** **Study design**

		**3 days after transplantation**		**3 weeks after transplantation**	
		**Control**	**VEGF**_**165**_	**Control**	**VEGF**_**165**_
***Number of mice***	*Grafted mice*	16	16	16	18
	*Dead mice*	2	1	1	0
	*Analysed grafted mice*	14	15	15	18
***IHC performed (data analysed)***	*H&E (follicular quantification and morphology evaluation)*			X	X
	*Dextran/FITC (functional blood vessels)*	X	X		
	*Dextran/FITC and α-SMA double staining (mature functional blood vessels)*	X	X		
	*Species-specific anti-mouse CD31*	X	X	X	X
	*Saffron (fibrosis)*			X	X

X: analysed; IHC: immunohistochemistry.

### Histological assessment

Each graft was embedded in paraffin and cut into 5-μm-thick serial sections. Several immunohistochemistries were performed after 3 days or 3 weeks of transplantation (Table 1). Ten sections per transplant, which covered the entire ovarian fragment, were stained with haematoxylin and eosin (H&E) and analysed as previously described to overcome the effect of heterogeneous follicular distribution within the transplanted ovarian cortex [24]. Only follicles in which a defined nucleus was visible were counted and separated into primordial, primary, secondary, or antral follicles according to the classification by Gougeon [31]. In these slices, follicular quality was evaluated and classified as normal or abnormal according to the morphological assessment. As previously described [32-34], the integrity of oocytes, granulosa cells, and basement membrane cells was observed. Follicles were classified as normal when they contained an intact oocyte and granulosa cells or abnormal when they contained a pyknotic oocyte nucleus and a shrunken ooplasm regardless of the presence of disorganised granulosa cells.

The immunohistochemical detection of vascular endothelial cells (CD31), functional blood vessels (dextran-FITC), and mature blood vessels (α-SMA) was performed using specific primary antibodies. Murine vascular blood vessel colonisation in the graft was detected using species-specific CD31 immunostaining. Incubation in PBS-BSA 10%, for 30 min at RT, was used to block non-specific binding sites. CD31 primary antibody (Ab) was diluted 1:100 in PBS-BSA 1% and incubated for 2 hours (h) at RT (Rat anti-mouse, ab56299, Abcam, Cambridge, United Kingdom), followed by incubation with the secondary Ab/HRP (AffiniPure rabbit anti-rat IgG diluted 1:4,000), (JAC-31200504, Lucron ELITechGroup, Puteaux, France) for 30 min at RT. Then, the reaction was revealed using DAB+ (K3468, Dako, Glostrup, Danemark).

Through the Dextran-FITC injection given to mice before sacrificing them, functional blood vessels were observed after immunostaining using an anti-fluorescein Ab (Converter-POD, Roche, Penzberg, Germany). Incubation in PBS-BSA 10%, for 30 min at RT, was used to block non-specific binding sites. This immunostaining was performed in one step by using an anti-fluorescein Ab/HRP ready to use (incubated for 30 min at RT), which directly recognised the FITC molecule fixed on Dextran injected before sacrifice, and the reaction was revealed using DAB+.

Mature functional blood vessels were recognised by double staining with dextran-FITC and α-SMA Abs. Incubation in PBS-BSA 10%, for 30 min at RT, was used to block non-specific binding sites. Staining against dextran-FITC was performed using an anti-fluorescein Ab/HRP ready to use (Converter-POD, Roche, Penzberg, Germany), which was incubated for 30 min at RT, and the reaction was revealed using DAB+. A second non-specific binding sites was blocked, by incubation in PBS-BSA 10% for 30 min at RT, before α-SMA staining was performed using a mouse anti-αSMA/FITC Ab diluted 1:1,000 in PBS-BSA 1% and incubated for 1 h 30 min at RT (F3777, Sigma-Aldrich, Saint Louis, MO, USA), followed by application of the AEC substrate chromogen (K3464, Dako, Glostrup, Danemark). In this double staining procedure, mature functional blood vessels appeared in red (α-SMA) and brown (dextran-FITC).

Fibrosis was evaluated using saffron staining. Incubation was performed for 10 min at 37°C in 1% saffron solution (Microm Microtech, Francheville, France) in absolute ethanol.

### Section and fibrosis analysis

Images of whole-tissue sections were digitised at high magnification (100×) to produce virtual images in which the pixel size was 1.510 μm. Vessels and the contour of the whole ovarian grafted tissue were drawn manually for each section. Then, the number of vessels and the surface of the whole tissue were measured automatically. Finally, the number of vessels was determined according to the surface units of ovarian grafted tissue. The image analysis was conducted using MATLAB 7.9 software.

Fibrosis was analysed on the digital images of saffron-stained slices using ImageJ software (National Institutes of Health, USA). Digitised colour images were first decomposed into three components of red, green, and blue. To enhance the features of fibrosis, the blue component was subtracted from the red component. The resulting images were then binarised using an appropriate threshold. With this method, pixels that were associated with fibrosis were assigned a value of 1 and pixels that were associated with the tissue were given a value of 0. The ratio between the surface that was occupied by the fibrotic area and the total surface area of the tissue represented the proportion of fibrotic tissue in each transplant.

### Statistical analyses

A statistical analysis was performed using GraphPad Prism software. The Mann–Whitney test was applied for comparisons between the two different groups. The statistical evaluation of the presence of blood vessels (dextran-FITC- or dextran-FITC/α-SMA-positive vessels) and the percentage of morphologically normal or abnormal follicles between the groups was performed using the Fisher’s exact test. Statistical significance was set at *p* ≤ 0.05.

## Results

### Vascularisation of ovarian cortical grafts

**Figure 2:**
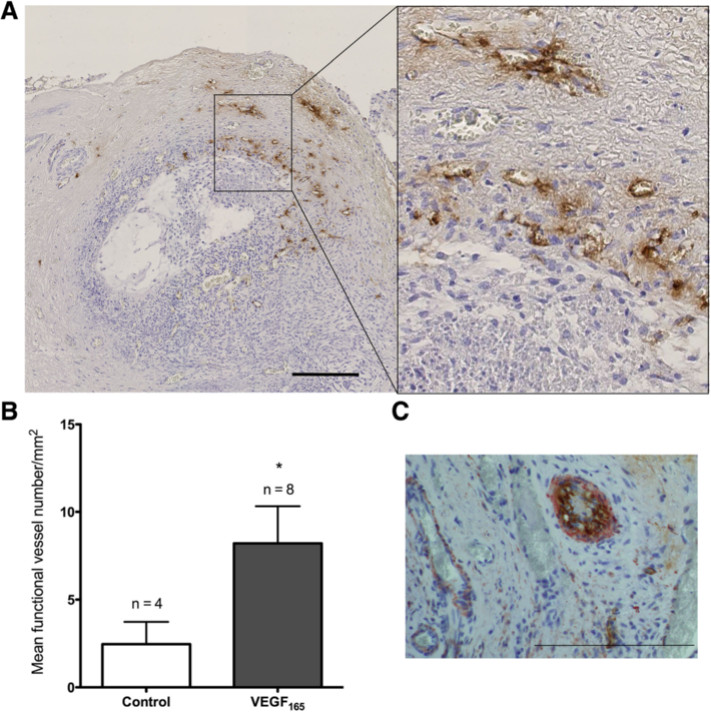
**The analysis of functional blood vessels in ovarian grafts 3 days after transplantation. A**. An illustration of functional blood vessels that were observed using dextran-FITC immunostaining. **B**. The quantification of FITC-positive vessels in ovarian transplants from the control and VEGF_165_-treated groups. In the treated mice, 8 of 15 fragments contained functional vessels compared with only 4 of 14 fragments in the control mice. **C**. An illustration of double staining for functional and mature blood vessels in a transplant that was treated with VEGF_165_. FITC was stained brown, and α-SMA was stained red. n corresponds to the number of transplanted ovarian fragments that contained functional blood vessels. Scale bar: 200 μm. *Corresponds to a *p* value < 0.05. Error bar are SEM (mean standard error).

Vascularisation in ovarian grafts was evaluated following an intravenous injection of dextran-fluorescein isothiocyanate (FITC) solution 3 min before sacrifice. Functional blood vessels in the ovarian transplants were detected after immunohistochemical labelling with FITC (Figure 2A).

#### Three days after transplantation

Functional (dextran-positive) blood vessels were detected in 53% of the mice (8/15) in the VEGF_165_-treated group versus 28.6% of the mice in the control group (4/14) (p = 0.26) (Table 2). In these FITC-positive transplants, functional blood vessel density was determined using semi-automated computer-assisted analysis as previously described [35]. A higher density of dextran-FITC-positive vessels was observed in the VEGF_165_ group (8.22 ± 2.10 vessels/mm^2^) compared with the control group (2.45 ± 1.27 vessels/mm^2^) (*p* = 0.03) (Figure 2B). To determine whether these functional vessels were mature, double immunostaining against dextran-FITC and alpha-smooth muscle actin (α-SMA) was performed to detect pericytes and/or smooth muscle cells (Figure 2C). Among the FITC-positive xenograft transplants (4/14 in the control group and 8/15 in the VEGF_165_-treated group), 50% of the transplants in the VEGF_165_-treated group were positive for α-SMA (4/8). In the control group, no transplants displayed double positivity (0/4) (*p* = 0.21) (Table 2). Double positivity demonstrated anastomosis between murine and ovine blood vessels, as murine neovessels within the fragment cannot be mature after only 3 days.

**Table 2:** **Number of ovarian transplants showing functional, mature functional and murine blood vessels 3 days after transplantation**

**Blood vessels**	**Control mice**	**VEGF**_**165**_ **50 nM treated mice**	***p*** **value**
*Dextran/FITC positive*	4/14 (28.6%)	8/15 (53%)	0.26
*Dextran/FITC and α-SMA Positive*	0/4 (0%)	4/8 (50%)	0.21
*CD31 positive*	3/14 (21.4%)	6/15 (40%)	0.43

Percentage of ovarian transplants showing functional blood vessels (dextran/FITC positive), mature functional blood vessels (double staining dextran/FITC and α-SMA positive) and murine blood vessels (CD31 positive) 3 days after transplantation in the VEGF_165_ treated group and control group.

Significance was established at a *p* value < 0.05.

**Figure 3:**
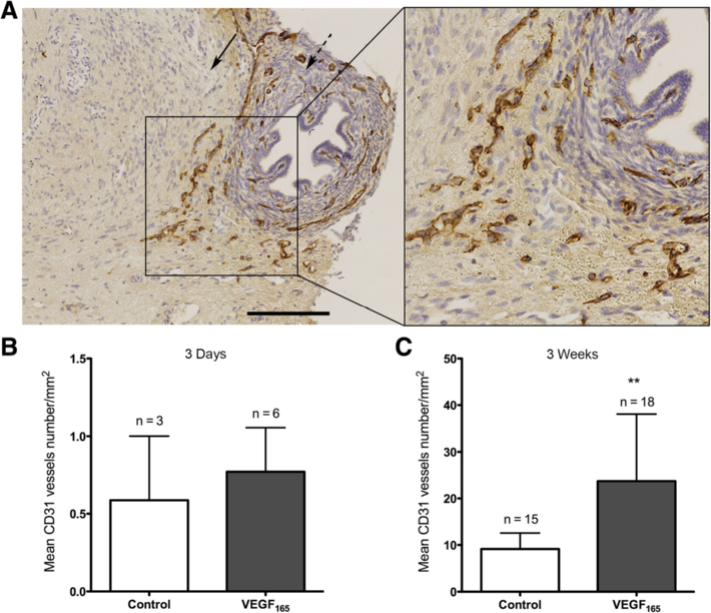
**- Murine blood vessels in sheep ovarian transplants after 3 days and 3 weeks reflect colonisation by host vessels into the graft. A**. An illustration of murine blood vessels that were stained with a mouse-specific CD31 Ab in an ovarian fragment that was treated with VEGF_165_ and removed 3 days after transplantation. The host tissue is identified by the dotted arrow, and the graft is identified by the plain arrow. **B**. The quantification of murine blood vessels in the grafted tissue 3 days after transplantation. In the treated mice, 6 of 15 fragments contained CD31-positive blood vessels compared with only 3 of 14 fragments in the control mice. **C**. The quantification of murine blood vessels in the grafted tissue 3 weeks after transplantation. All of the fragments in both the control and treated groups contained CD31-positive blood vessels. n corresponds to the number of transplanted ovarian fragments that contained murine blood vessels. Scale bar: 200 μm. **Corresponds to a *p* value < 0.01. Error bar are SEM.

As previously described, vascularisation of xenografted ovarian tissue results from anastomoses between ovarian vessels and host vessels, as demonstrated through previous double staining, and from graft colonisation by murine blood vessels, termed chimeric vessels [36]. The immunodetection of murine CD31 using a species-specific anti-mouse CD31 antibody (Ab) identified blood vessels that had migrated from the murine uterine horn to the sheep ovarian graft 3 days post-transplantation (Figure 3A). A higher proportion of ovarian grafts that contained murine-specific CD31-positive vessels were observed in the VEGF_165_-treated group (40%, n = 15) compared with the untreated group (21.4%, n = 14), but the difference was not significant (*p* = 0.43) (Table 2). However, no difference in vessel density was observed between the two experimental groups (0.77 ± 0.28 vessels/mm^2^ in the VEGF_165_-treated group versus 0.59 ± 0.41 vessels/mm^2^ in the control group, p = 0.36) (Figure 3B).

#### Three weeks after transplantation

In contrast, 3 weeks after grafting, a higher density of species-specific murine CD31-positive vessels was observed in the VEGF_165_ group (23.72 ± 14.36 vessels/mm^2^, n = 18) compared with the control group (9.13 ± 3.45 vessels/mm^2^, n = 15) (*p* < 0.01) (Figure 3C).

### Fibrosis, follicular density, and morphology

**Figure 4:**
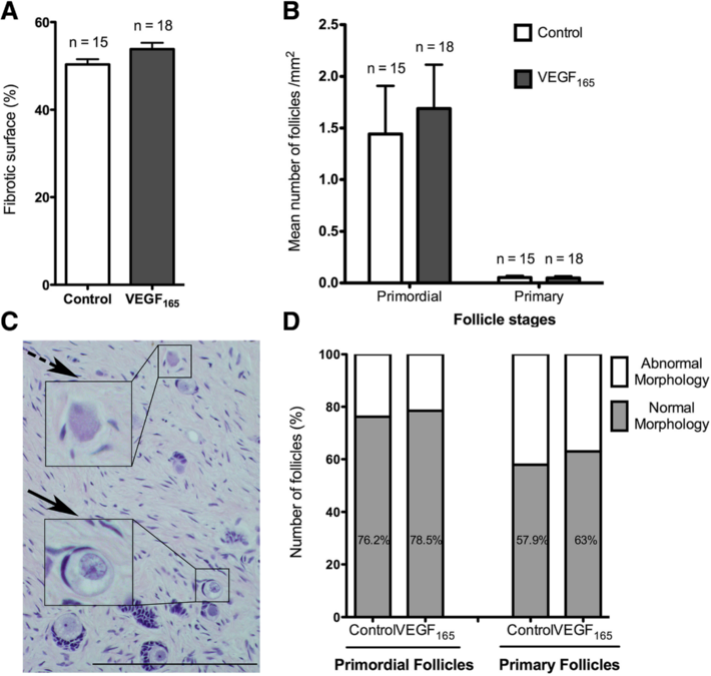
**The analysis of fibrosis and follicles 3 weeks after transplantation. A**. The percentage of fibrotic tissue in the ovarian transplant. **B**. Primordial and primary follicle quantification in H&E-stained sections (mean number/mm^2^). **C**. An illustration of primordial follicles that were considered to be morphologically normal (the plain arrow) or abnormal (the dotted arrow) with disorganised granulosa cells. **D**. The proportion of morphologically normal and abnormal primordial and primary follicles in the control group and the VEGF_165_-treated group. n corresponds to the number of ovarian fragments that were transplanted into mice. Scale bar: 200 μm. Error bar are SEM.

Analysis of fibrosis, evaluated using saffron staining, showed a similar extent of fibrosis in the VEGF_165_ (53.82 ± 1.45%, n = 18) and control (50.31 ± 1.2%, n = 15) groups three weeks after grafting (*p* = 0.053) (Figure 4A). The analysis of primordial and primary follicle density in haematoxylin and eosin (H&E)-stained sections 3 weeks after grafting indicated a predominance of primordial follicles in the graft (Figure 4B). However, no differences between the treated and untreated groups in the follicle density of primordial (1.69 ± 0.42 and 1.44 ± 0.47 follicles/mm^2^, respectively, with a total of 2349 follicles analysed, *p* = 0.33) and primary follicles (0.05 ± 0.02 and 0.05 ± 0.02 follicles/mm^2^, respectively, with a total of 65 follicles analysed, *p* = 0.32) (Figure 4B) or morphological anomalies of primordial (78.46 and 76.20% of morphologically normal follicles, respectively, respectively) and primary follicles (62.96 and 57.90% of morphologically normal follicles, respectively) (Figures 4C-D) were observed. In addition, the low density of secondary and more mature follicles prevented further statistical analysis.

## Discussion

In this study, the effect of VEGF_165_ on slow-frozen, cryopreserved ewe ovarian tissue that was grafted into a murine model was analysed three days and three weeks after transplantation. The results of this study suggest that VEGF_165_ improves angiogenesis in ovarian grafts as soon as three days post-transplantation. In addition, this treatment enhanced vessel maturation because 50% of the ovarian transplants that were impregnated with VEGF_165_ exhibited mature vessels that were surrounded by alpha-smooth muscle cells. This rapid reconstruction of blood vessels in ovarian grafts is important for limiting ischemic injury, which has been demonstrated to induce the depletion of 60-95% of the follicular reserve, including the loss of virtually the entire population of growing follicles, during autograft processes [7,37-39]. This phenomenon has been associated with a dramatic reduction in graft size and significant fibrosis [40]. In this study, three weeks after transplantation, the extent of fibrosis was similar between the control and VEGF_165_-treated groups. We previously demonstrated that fibrosis that occurred three weeks after transplantation was partially due to the freeze-thawing procedure [8]. Therefore, more rapid revascularisation of the transplant after grafting due to the pro-angiogenic effect of VEGF_165_ was not likely to limit the development of fibrosis.

This study analysed ovarian fragments that were encapsulated in a collagen gel that contained VEGF_165._ Indeed, given the short half-life time of VEGF and in order to have a longer local delivery, a matrix containing VEGF was necessary. In our laboratory, encapsulation of ovarian fragment in a collagen gel had already been developed and proved efficacious. However, other teams have also study the release of VEGF in different models like alginate scaffold [41] or fibrin matrix [42-45]. In this study, the results regarding vascular improvement are in agreement with those from studies that used different systems, such as the encapsulation of ovarian fragments in a VEGF_168_ fibrin matrix and autologous murine transplantation [42-45]. Additionally, the combined effect of VEGF and basic fibroblast growth factor (bFGF) was found to increase graft survival in an experimental rabbit model of xenografted human ovarian tissue by triggering angiogenesis and by reducing apoptosis and fibrosis [45]. The present study confirmed that VEGF_165_ triggers the angiogenic process; however, VEGF_165_ did not reduce fibrosis or affect follicle survival.

The pre-existing vasculature can initiate the formation of new blood vessels either by sprouting, intussusception, or elongation via the incorporation of circulating endothelial cells [46]. In the context of the physiological angiogenic process of folliculogenesis, most of these processes are effective [47]. Ovarian tissue grafts are exposed to ischemic damage during the post-transplantation period until the vasculature develops. Vascular connections between the host and an ovarian strip that was grafted into a murine ovary were observed 5 days after transplantation [13]. To determine whether the addition of VEGF_165_ had a beneficial effect on vascular recruitment and limited the period of tissue hypoxia, transplanted fragments were analysed three days and three weeks after grafting. The analysis of vascularisation revealed that VEGF_165_ effectively improved graft vascularisation as early as three days post-transplantation. Moreover, a higher proportion of functional blood vessels (dextran FITC-positive vessels) and mature vessels (α-SMA) was observed. Using species-specific CD31 immunohistochemistry and double staining for functional and mature blood vessels (Dextran-FITC and α-SMA), we demonstrated the dual origin of angiogenesis (from the host and from the grafted tissue), as previously described [36]. Collectively, these data confirm the pro-angiogenic effect of VEGF_165_ and the presence of murine endothelial cell migration towards the sheep transplant. However, this improved vascularisation did not modulate the follicular density and did not alter the follicular morphology. In this study, follicular morphology was evaluated in several sections using light microscopy scoring as previously described [24]. VEGF_165_ was used in this study, following our encouraging results with VEGF_111_, because it offers the advantage, with respect to the 111 isoform, of existing both in free and matrix-bound forms, which could improve its release profile from the collagen matrix. However, in contrast to VEGF_111_, which has been demonstrated to preserve primary ovarian follicles and improve angiogenesis [23], VEGF_165_ failed to significantly decrease fibrosis or increase oocyte survival and morphology. Various VEGF isoforms have exhibited differences in mitogenicity, chemotactic efficacy, receptor interaction, and tissue distribution [48,49]. However, a direct comparison of VEGF_111_ and VEGF_165_ in terms of the follicular pool, survival, and morphology preservation has yet to be performed. Another key factor that affects the efficacy of cytokine treatments is the route of administration of these molecules. Skaznik-Wikiel et al. previously reported that intraperitoneal injections of VEGF_164_ for five days combined with subcutaneous injections of granulocyte colony-stimulating factor (G-CSF) maintained the primordial follicular pool [43]. In addition, Friedman et al. found that graft incubation with hyaluronan-rich biological glue combined with VEGF-A and vitamin E decreased the number of atretic follicles and the levels of apoptosis two weeks after grafting in a xenograft model [44]. Additionally, a recent study demonstrated that human ovarian graft incubation followed by hypodermic injections at the transplant site with a combination of VEGF and bFGF 7 days after transplantation improved angiogenesis and reduced apoptosis and fibrosis 6 weeks after transplantation [45]. Although these studies have demonstrated the beneficial effects of VEGF, they often used a method of systemic delivery of VEGF, which is not reasonable in the context of clinical usage in patients with cancer. The major advantage of our model was limiting local exposure to VEGF to the site of transplantation. Other molecules used without VEGF such as S1P [15], EPO [16], or PRP [17] have been shown to improve the quality of ovarian grafts and could therefore be interesting to study in combination with VEGF. Other molecules such as human menopausal gonadotropin (HMG) act indirectly, increasing endogenous VEGF secretion in a transplant after three hours of culture before grafting*.* HMG treatment accelerated revascularisation of the transplant and increased the number of surviving follicles one month after transplantation [50]. These data emphasise the potential benefits of combining different growth factors to prevent early ischemic damage, improve rapid revascularisation of the transplant, and maintain the follicular pool.

## Conclusions

This study demonstrates that encapsulation of ovarian tissue with VEGF_165_ in a collagen matrix during xenografting in mice produces a more rapid onset of functional vessel formation and earlier revascularisation of the transplant. Collectively, our data suggest that the prevention of hypoxia during ovarian tissue grafting requires additional cofactors to preserve the follicular pool.

## Abbreviations

α-SMA: Type alpha of smooth muscle actin
Ab(s): Antibody(ies)
AEC: 3-amino-9-ethylcarbazole
bFGF: Basic fibroblast growth factor
BSA: Bovine serum albumin
CD31: Cluster of differentiation 31
Col I: Collagen type I
DAB: 3,3′-diaminobenzidine
DMSO: Dimethyl sulfoxide
EPO: Erythropoietin
FITC: Fluorescein isothiocyanate
G-CSF: Granulocyte colony stimulating factor
H: Hour
HMG: Human menopausal gonadotrophin
HRP: Horse radish peroxidase
H&E: Hematoxylin & eosin
IHC: Immunohistochemistry
Min: Minutes
P: p-value
PBS: Phosphate buffered saline
POF: Premature ovarian failure
PRP: Platelet-rich plasma
RT: Room temperature
SCID mice: Severe combined immunodeficient mice
S1P: Sphingosine-1-phosphate
VEGF-R: VEGF-receptor
VEGF: Vascular endothelial growth factor

## References

[1] Maltaris T, Seufert R, Fischl F, Schaffrath M, Pollow K, Koelbl H (2007). The effect of cancer treatment on female fertility and strategies for preserving fertility. Eur J Obstet Gynecol Reprod Biol.

[2] Dillon KE, Gracia CR (2012). Pediatric and young adult patients and oncofertility. Curr Treat Options Oncol.

[3] Green DM, Kawashima T, Stovall M, Leisenring W, Sklar CA, Mertens AC (2009). Fertility of female survivors of childhood cancer: a report from the childhood cancer survivor study. J Clin Oncol.

[4] West ER, Zelinski MB, Kondapalli LA, Gracia C, Chang J, Coutifaris C (2009). Preserving female fertility following cancer treatment: current options and future possibilities. Pediatr Blood Cancer.

[5] Donnez J, Dolmans MM, Pellicer A, Diaz-Garcia C, Sanchez Serrano M, Schmidt KT (2013). Restoration of ovarian activity and pregnancy after transplantation of cryopreserved ovarian tissue: a review of 60 cases of reimplantation. Fertil Steril.

[6] Donnez J, Dolmans MM (2013). Fertility preservation in women. Nat Rev Endocrinol.

[7] Baird DT, Webb R, Campbell BK, Harkness LM, Gosden RG (1999). Long-term ovarian function in sheep after ovariectomy and transplantation of autografts stored at −196 C. Endocrinology.

[8] Nisolle M, Casanas-Roux F, Qu J, Motta P, Donnez J (2000). Histologic and ultrastructural evaluation of fresh and frozen-thawed human ovarian xenografts in nude mice. Fertil Steril.

[9] Nichols-Burns SM, Lotz L, Schneider H, Adamek E, Daniel C, Stief A (2014). Preliminary observations on whole-ovary xenotransplantation as an experimental model for fertility preservation. Reprod Biomed Online.

[10] Maffei S, Hanenberg M, Pennarossa G, Silva JR, Brevini TA, Arav A (2013). Direct comparative analysis of conventional and directional freezing for the cryopreservation of whole ovaries. Fertil Steril.

[11] Nugent D, Newton H, Gallivan L, Gosden RG (1998). Protective effect of vitamin E on ischaemia-reperfusion injury in ovarian grafts. J Reprod Fertil.

[12] Martinez-Madrid B, Donnez J, Van Eyck AS, Veiga-Lopez A, Dolmans MM, Van Langendonckt A (2009). Chick embryo chorioallantoic membrane (CAM) model: a useful tool to study short-term transplantation of cryopreserved human ovarian tissue. Fertil Steril.

[13] Van Eyck AS, Jordan BF, Gallez B, Heilier JF, Van Langendonckt A, Donnez J (2009). Electron paramagnetic resonance as a tool to evaluate human ovarian tissue reoxygenation after xenografting. Fertil Steril.

[14] David A, Van Langendonckt A, Gilliaux S, Dolmans MM, Donnez J, Amorim CA (2012). Effect of cryopreservation and transplantation on the expression of kit ligand and anti-Mullerian hormone in human ovarian tissue. Hum Reprod.

[15] Soleimani R, Heytens E, Oktay K (2011). Enhancement of neoangiogenesis and follicle survival by sphingosine-1-phosphate in human ovarian tissue xenotransplants. PLoS One.

[16] Commin L, Buff S, Rosset E, Galet C, Allard A, Bruyere P (2012). Follicle development in cryopreserved bitch ovarian tissue grafted to immunodeficient mouse. Reprod Fertil Dev.

[17] Callejo J, Salvador C, Gonzalez-Nunez S, Almeida L, Rodriguez L, Marques L (2013). Live birth in a woman without ovaries after autograft of frozen-thawed ovarian tissue combined with growth factors. J Ovarian Res.

[18] Abir R, Ao A, Zhang XY, Garor R, Nitke S, Fisch B (2010). Vascular endothelial growth factor A and its two receptors in human preantral follicles from fetuses, girls, and women. Fertil Steril.

[19] Dvorak HF (2000). VPF/VEGF and the angiogenic response. Semin Perinatol.

[20] Mineur P, Colige AC, Deroanne CF, Dubail J, Kesteloot F, Habraken Y (2007). Newly identified biologically active and proteolysis-resistant VEGF-A isoform VEGF111 is induced by genotoxic agents. J Cell Biol.

[21] Park JE, Keller GA, Ferrara N (1993). The vascular endothelial growth factor (VEGF) isoforms: differential deposition into the subepithelial extracellular matrix and bioactivity of extracellular matrix-bound VEGF. Mol Biol Cell.

[22] Robinson CJ, Stringer SE (2001). The splice variants of vascular endothelial growth factor (VEGF) and their receptors. J Cell Sci.

[23] Labied S, Delforge Y, Munaut C, Blacher S, Colige A, Delcombel R (2013). Isoform 111 of vascular endothelial growth factor (VEGF111) improves angiogenesis of ovarian tissue xenotransplantation. Transplantation.

[24] Fransolet M, Labied S, Henry L, Masereel MC, Rozet E, Kirschvink N (2014). Strategies for using the sheep ovarian cortex as a model in reproductive medicine. PLoS One.

[25] Gosden RG, Baird DT, Wade JC, Webb R (1994). Restoration of fertility to oophorectomized sheep by ovarian autografts stored at −196 degrees C. Hum Reprod.

[26] Lornage J, Courbiere B, Mazoyer C, Odagescu V, Baudot A, Bordes A (2006). Ovarian tissue vitrification: cortex and whole ovary in sheep. Gynecol Obstet Fertil.

[27] Courbiere B, Massardier J, Salle B, Mazoyer C, Guerin JF, Lornage J (2005). Follicular viability and histological assessment after cryopreservation of whole sheep ovaries with vascular pedicle by vitrification. Fertil Steril.

[28] Sauvat F, Bouilly J, Capito C, Lefevre A, Blachere T, Borenstein N (2013). Ovarian function is restored after grafting of cryopreserved immature ovary in ewes. FASEB J.

[29] Gosden RG, Boulton MI, Grant K, Webb R (1994). Follicular development from ovarian xenografts in SCID mice. J Reprod Fertil.

[30] Fusenig NE, Limat A, Stark HJ, Breitkreutz D (1994). Modulation of the differentiated phenotype of keratinocytes of the hair follicle and from epidermis. J Dermatol Sci.

[31] Gougeon A (1986). Dynamics of follicular growth in the human: a model from preliminary results. Hum Reprod.

[32] Faustino LR, Santos RR, Silva CM, Pinto LC, Celestino JJ, Campello CC (2010). Goat and sheep ovarian tissue cryopreservation: effects on the morphology and development of primordial follicles and density of stromal cell. Anim Reprod Sci.

[33] David A, Dolmans MM, Van Langendonckt A, Donnez J, Amorim CA (2011). Immunohistochemical localization of growth factors after cryopreservation and 3 weeks’ xenotransplantation of human ovarian tissue. Fertil Steril.

[34] Sanfilippo S, Canis M, Romero S, Sion B, Dechelotte P, Pouly JL (2013). Quality and functionality of human ovarian tissue after cryopreservation using an original slow freezing procedure. J Assist Reprod Genet.

[35] Balsat C, Blacher S, Signolle N, Beliard A, Munaut C, Goffin F (2011). Whole slide quantification of stromal lymphatic vessel distribution and peritumoral lymphatic vessel density in early invasive cervical cancer: a method description. ISRN Obstet Gynecol.

[36] Van Eyck AS, Bouzin C, Feron O, Romeu L, Van Langendonckt A, Donnez J (2010). Both host and graft vessels contribute to revascularization of xenografted human ovarian tissue in a murine model. Fertil Steril.

[37] Aubard Y, Piver P, Cogni Y, Fermeaux V, Poulin N, Driancourt MA (1999). Orthotopic and heterotopic autografts of frozen-thawed ovarian cortex in sheep. Hum Reprod.

[38] Liu L, Wood GA, Morikawa L, Ayearst R, Fleming C, McKerlie C (2008). Restoration of fertility by orthotopic transplantation of frozen adult mouse ovaries. Hum Reprod.

[39] Aubard Y (2003). Ovarian tissue xenografting. Eur J Obstet Gynecol Reprod Biol.

[40] Kim SS, Soules MR, Battaglia DE (2002). Follicular development, ovulation, and corpus luteum formation in cryopreserved human ovarian tissue after xenotransplantation. Fertil Steril.

[41] Kedem A, Perets A, Gamlieli-Bonshtein I, Dvir-Ginzberg M, Mizrahi S, Cohen S (2005). Vascular endothelial growth factor-releasing scaffolds enhance vascularization and engraftment of hepatocytes transplanted on liver lobes. Tissue Eng.

[42] Shikanov A, Zhang Z, Xu M, Smith RM, Rajan A, Woodruff TK (2011). Fibrin encapsulation and vascular endothelial growth factor delivery promotes ovarian graft survival in mice. Tissue Eng Part A.

[43] Skaznik-Wikiel ME, Sharma RK, Selesniemi K, Lee HJ, Tilly JL, Falcone T (2011). Granulocyte colony-stimulating factor in conjunction with vascular endothelial growth factor maintains primordial follicle numbers in transplanted mouse ovaries. Fertil Steril.

[44] Friedman O, Orvieto R, Fisch B, Felz C, Freud E, Ben-Haroush A (2012). Possible improvements in human ovarian grafting by various host and graft treatments. Hum Reprod.

[45] Wang L, Ying YF, Ouyang YL, Wang JF, Xu J (2013). VEGF and bFGF increase survival of xenografted human ovarian tissue in an experimental rabbit model. J Assist Reprod Genet.

[46] Yancopoulos GD, Davis S, Gale NW, Rudge JS, Wiegand SJ, Holash J (2000). Vascular-specific growth factors and blood vessel formation. Nature.

[47] Macchiarelli G, Jiang JY, Nottola SA, Sato E (2006). Morphological patterns of angiogenesis in ovarian follicle capillary networks. A scanning electron microscopy study of corrosion cast. Microsc Res Tech.

[48] Keyt BA, Berleau LT, Nguyen HV, Chen H, Heinsohn H, Vandlen R (1996). The carboxyl-terminal domain (111–165) of vascular endothelial growth factor is critical for its mitogenic potency. J Biol Chem.

[49] Neufeld G, Cohen T, Gitay-Goren H, Poltorak Z, Tessler S, Sharon R (1996). Similarities and differences between the vascular endothelial growth factor (VEGF) splice variants. Cancer Metastasis Rev.

[50] Wang Y, Chang Q, Sun J, Dang L, Ma W, Hei C (2012). Effects of HMG on revascularization and follicular survival in heterotopic autotransplants of mouse ovarian tissue. Reprod Biomed Online.

